# Compositional analysis of topsoil metals and its associations with cancer mortality using spatial misaligned data

**DOI:** 10.1007/s10653-016-9904-3

**Published:** 2017-02-02

**Authors:** Gonzalo López-Abente, Juan Locutura-Rupérez, Pablo Fernández-Navarro, Iván Martín-Méndez, Alejandro Bel-Lan, Olivier Núñez

**Affiliations:** 1 Environmental and Cancer Epidemiology Unit, National Centre for Epidemiology, Carlos III Institute of Health, Avda. Monforte de Lemos 5, 28029 Madrid, Spain; 2 Consortium for Biomedical Research in Epidemiology and Public Health (CIBER en Epidemiología y Salud Pública - CIBERESP), Madrid, Spain; 3 Department of Geochemistry and Mineral Resources, Spanish Geological and Mining Institute (Instituto Geológico y Minero de España/IGME), Ríos Rosas, 23, 28003 Madrid, Spain

**Keywords:** Cancer mortality, Spatial data analysis, Geochemistry, Soil composition, Compositional analysis, Medical geology

## Abstract

The presence of toxic metals in soil per se, and in soil impacted by mining, industry, agriculture and urbanisation in particular, is a major concern for both human health and ecotoxicology. The dual aim of this study was: to ascertain whether topsoil composition could influence the spatial distribution of mortality due to different types of cancer and to identify possible errors committed by epidemiological studies which analyse soil composition data as a closed number system. We conducted an ecological cancer mortality study, covering 861,440 cancer deaths (27 cancer sites) in 7917 Spanish mainland towns, from 1999 to 2008. Topsoil levels of Al, As, Cd, Cr, Cu, Fe, Mn, Ni, Pb and Zn were determined by ICP-MS at 13,317 sampling points. We transformed the topsoil data in two ways, i.e. log transformation and centred logratio transformation. Principal factor analysis was performed to obtain independent latent factors for the transformed variables. To estimate the effect on mortality of topsoil factor loadings, we fitted Besag, York and Mollié models embedded in geostatistical-spatial models. This model included soil sample locations and town centroids (non-aligned data), fitted using the integrated nested Laplace approximation (INLA) as a tool for Bayesian inference and stochastic partial differential equations (SPDE). All results were adjusted for socio-demographic variables. The results indicated that soil composition could have an influence on the spatial distribution and mortality patterns of cancer. The analysis adjusted for socio-demographic variables showed excess male mortality due to digestive system tumours in areas with soils containing higher Cd, Pb, Zn, Mn and Cu concentrations, bladder cancer in areas with soils containing higher Cd concentrations, and brain cancer in areas with soils containing As. In both sexes, cancer of oesophagus was associated with soils containing a higher lead content, while lung cancer was associated with soils containing a higher copper content. Stress should be laid on the importance of taking into account the compositional nature of the data in this type of analysis.

**Electronic supplementary material:**

The online version of this article (doi:10.1007/s10653-016-9904-3) contains supplementary material, which is available to authorized users.

## Introduction

Chemical composition of soil, due to its geological origin, remains stable over time and can contain carcinogens such as heavy metals. In theory, human exposure to bioavailable carcinogenic components of soil can affect both sexes indiscriminately. On the other side, spatio-temporal cancer mortality studies and various cancer atlases in Spain have revealed geographical patterns for some tumours, which display similar spatial distribution in men and women and persist over time (López-Abente et al. 2007, 2014). Furthermore, the determinants of these patterns have been very difficult to ascertain. Cancers of the upper gastrointestinal tract (stomach and oesophagus), pancreas, brain, kidney and thyroid all display the above characteristics.

The presence of toxic metals in soil per se, and in soil impacted by human activity in particular (Fernández-Navarro et al. 2012; García-Pérez et al. 2007; García-Perez et al. 2016), is a major concern for both human health and ecotoxicology (Ranville 2005). High-level exposures to arsenic and heavy metals have been found to be associated with multiple cancer types by numerous epidemiological studies (Naujokas et al. 2013). There is far less information, however, on the health effects of low-dose chronic exposure to many trace metals, and studies on the health effects of metals and metalloids in topsoil belong to this latter category (Centeno et al. 2013).

Data drawn from geochemical soil studies are recorded in mg/kg or parts per million (ppm) and come within the category of compositional data or closed number systems (Aitchison 1994, Reimann et al. 2011). Data of this type are vectors whose components are the proportion of some whole and are thus not independent (e.g. their sum is some constant). For instance, in geochemical soil studies, this could induce negative correlations or no correlations in variables one would logically expect to be positively correlated. The usual approach in compositional analysis is to resort to different transformations (logratio analysis) (Aitchison 1982), removing the closure effects in data points. Another difficulty of these analyses is that, depending upon the local geological formation, there will be one or more naturally associated soil elements (Selinus et al. 2013). The results of individual, isolated analysis of some components may thus raise doubts as to the associations found. The usual form of analysis is to study the possible effect of the soil element associations detected, by means of factorial analysis.

The development of environmental monitoring networks and advances in sensor technology make for a data-rich environment that affords extraordinary opportunities for understanding the complexity of geocoded ecological data (Finley et al. 2014). In ecological analysis, one usually tries to draw inferences about an association between multiple variables or to predict their values at new locations. The study reported here sought to use data on topsoil composition in a grid of 13,000 points to ascertain the influence of local soil composition on the distribution of cancer mortality in >8000 towns. In the literature, this is known as point areal spatial misalignment (Finley et al. 2014), though there are authors who prefer the term, “change of support problem” (Gotway and Young 2002).

To deal with the misalignment issue, exposure (in this case, topsoil metal composition) can be predicted at the outcome location using spatial interpolation methods (kriging). The uncertainty surrounding such exposure (kriging error) is by no means negligible and may vary substantially from one area to another. A naïve inference ignores this error, something that may in turn lead to a highly biased estimate of the exposure effect and poor coverage of the confidence interval (Gryparis et al. 2009). Yet, if the inference is performed in a model where spatial variations of the exposure and the health outcome are jointly modelled, any uncertainty associated with the exposure prediction is implicitly taken into account (Blangiardo and Cameletti 2015). However, this measurement error model approach is computationally expensive if all metal composition components are included in the model. The factor analysis suggested above to solve the multicollinearity problem can also notably reduce this calculation cost. The factors extracted from this analysis are, by construction, mutually independent. Hence, analysis of the association between cancer mortality risk and topsoil metal composition can be broken down into a few covariates of one-dimensional exposure association analyses.

Accordingly, this study sought to answer the following questions: (1) could topsoil composition influence the spatial distribution of mortality at different cancer sites? and (2) which errors are committed when soil composition data are analysed as a closed number system in epidemiological studies?

## Materials and methods

### Mortality data

A detailed description of the mortality data and soil samples collected can be found in a previously published study (Núñez et al. 2016). Briefly, mortality data (observed and expected cases) for each of the 8077 (7917 mainland) Spanish towns were drawn from the records of the National Statistics Institute (NSI) for the study period (1999–2008) and computed for 13 types of malignant tumours (see Supplementary data, Table S1), with a total of 669,973 deaths due to the tumours analysed.

### Soil sampling and metal analysis

Across the period June 2008–November 2010, a total of 21,187 residual soil samples (13,505 from the surface horizon and 7682 from the deeper horizon) were collected at a total of 13,505 sampling points (13,317 in mainland Spain and 188 on the Canary and Balearic islands). The residual soil samples were analysed by instrumental inductively coupled plasma mass spectrometry (ICP-MS). The elements included in this analysis were Al, As, Cd, Cr, Cu, Fe, Mn, Ni, Pb and Zn. A detailed description of the sample collection and the chemical analysis techniques used can be found in the Geochemical Atlas of Spain (Locutura et al. 2012). All the laboratory determinations were performed at Activation Laboratories Ltd. (Actlabs, Ontario).

### Topsoil data transformations

Topsoil data were transformed prior to their analysis in two ways, i.e. log transformation (classical option) and centred logratio transformation (clr transformation).

Log transformation consists of standardisation of the logarithm of the concentration:

$$y = \frac{{\left( {{ \log }\left( x \right) - {\text{mean}}\left( {{ \log }\left( x \right)} \right)} \right)}}{{\left( {{\text{sd}}\left( {{ \log }\left( x \right)} \right)} \right)}}$$

For compositional data, the sum of all concentrations of the elements in each sample is almost constant or at least restricted. To avoid spurious correlations, the soil compositions estimated for the respective towns were transformed by clr transformation (Filzmoser et al. 2010; Aitchison 1982, 2003). This results in a multivariate observation *y* = (*y*_1_,…, *y*_D_) and is defined as:

$$y = \left( {{ \log }\frac{{x_{1} }}{{\sqrt {\mathop \prod \nolimits_{i = 1}^{D} x_{i} } }} \ldots ,\;{ \log }\frac{{x_{D} }}{{\sqrt {\mathop \prod \nolimits_{i = 1}^{D} x_{i} } }}} \right)$$

Each value of a variable for each point was divided by the geometric mean of all variables for that point, and the logarithms then obtained. This is the preferable method for opening a data set whenever a direct relation to the variables is needed (Reimann et al. 2011).

### Reduction in dimensions (factorial analysis)

A direct consequence of data log transformation is emerging collinearity (e.g. cadmium occurs mainly in ores with zinc and, to a lesser degree, with lead and copper). Moreover, the clr transformation produces variables whose correlation matrix is singular. It is therefore difficult to perform a regression analysis with such explanatory variables. Moreover, the effects on human health might possibly derive from exposure to associations between elements, which would mean that such associations would be the variable of exposure of interest.

In order to avoid this problem, a factorial analysis was conducted to obtain independent latent factors for the log-transformed and clr-transformed variables. This type of analysis provides information about the internal structure of the geochemical data, reduces data dimensionality to a few representative factors and thus seeks to summarise the multivariate information in a compact manner. We performed this analysis by using principal factor analysis (PFA) (Reimann et al. 2011), in which a cumulative variance of over 75% was obtained by 4 factors for log-transformed data and 5 factors for clr-transformed data. For statistical analysis purposes, the factor scores for each topsoil sample point were extracted after rotation by the varimax method. Information on the output of principal components analysis for the two variable transformations is shown graphically on a biplot.

To make it easier to interpret the results of the analysis of the clr-transformed data, the sign of the factor loadings was changed, since the metals most representative of the factors had negative factor loadings.

#### Statistical analysis of spatially misaligned data

Cancer mortality data are aggregated at a town area level, while the data concentrations of elements in topsoil are measures taken at sampling locations across the country. To take this into account, we therefore adopted an approach whereby spatial variations in metal concentrations (topsoil sampling locations) and in relative risks of cancer mortality (town locations) were jointly modelled and estimated (spatially misaligned data).

Let ${\text{expos}}_{i}$ denote the topsoil composition indicator (factorial scores) at each centroid area location $s_{i}$ and assume for the moment that these values are known. We assume that the observed number of cases $O_{i}$ in the *i*th area is Poisson-distributed, with mean $E_{i} \lambda_{i}$, where $E_{i}$ is the expected number of cases in that area and the relative risk $\lambda_{i}$ follows a log-linear model, such that:

1 $${ \log }\left( {\lambda_{i} } \right) = \alpha \,+\, \beta {\text{expos}}_{i} \, +\, u_{i} \,+\, v_{i} ,$$

where $\alpha$ is an intercept, $\beta$ is the coefficient for the exposure covariate ${\text{expos}}_{i}$, $v_{i}$ are unstructured normal residuals, and $u_{i}$ are spatially structured effects which follow an intrinsic conditional autoregressive model, namely the Besag, York and Mollié model (BYM) (Besag et al. 1991). Inference for the primary parameter of interest $\beta$ is made in a Bayesian framework, and prior distributions are specified for all parameters.

In point of fact, the exposure covariate ${\text{expos}}_{i}$ is not directly observed. Instead, we observe the factor scores $c_{j}$ at sampling locations $s_{j}$. For these observations, we assume the log-linear model

2 $${ \log }\left( {c_{j} } \right){\text{ = Normal }}\left( {x_{j} ,\sigma_{x}^{2} } \right),$$

where $x_{j}$ is the realisation of a Matérn Gaussian field at location $s_{j}$ and $\sigma_{x}^{2}$ is a measurement error variance.

In our approach, ${\text{expos}}_{i}$ is a latent variable equal to $x_{i}$ and its relationship with the relative risk of mortality is assessed through joint estimation of models (1) and (2). Hence, this approach leads to conservative confidence intervals, as it takes into account the uncertainty in the exposure variable. The Gaussian field in model (2) was approximated using the stochastic partial differential equation (SPDE) (Lindgren et al. 2011; Lindgren and Rue 2015), as implemented in integrated nested Laplace approximation (R-INLA) (Rue et al. 2009; Rue and Martino 2010). This approach is based on a triangulated mesh of mainland Spain (Núñez et al. 2016). The choice of the mesh resolution (number of vertices) is a compromise between the accuracy of this approach and computational costs. To solve this trade-off, we used an information criterion based on the greatest length of the triangle edge allowed, with the selected value of this length being 5 km. The extension of the mesh with a lower resolution around the Spanish mainland was constructed to control for boundary effects.

In addition to model (1), another ecological regression (3) was considered to account for potential socio-demographic and environmental confounding factors:

3 $$\log \left( {\lambda_{i} } \right) = \alpha + \beta \exp{\text{os}}_{i} + \mathop \sum \limits_{j} \delta_{j} {\text{Soc}}_{ij} + u_{i} + v_{i} ,$$

where the socio-demographic indicators (${\text{Soc}}_{ij}$) were obtained from the 1991 census and considered for their availability at the city level and potential explanatory ability vis-à-vis certain geographical mortality patterns (López-Abente et al. 2006). These indicators (*j*) were: population size (categorised into three levels: 0–2000 [rural zone], 2000–10,000 [semi-urban zone] and greater than 10,000 inhabitants [urban zone]); percentages of illiteracy, farmers and unemployment; average number of persons per household; and mean income.

## Results

**Fig. 1:**
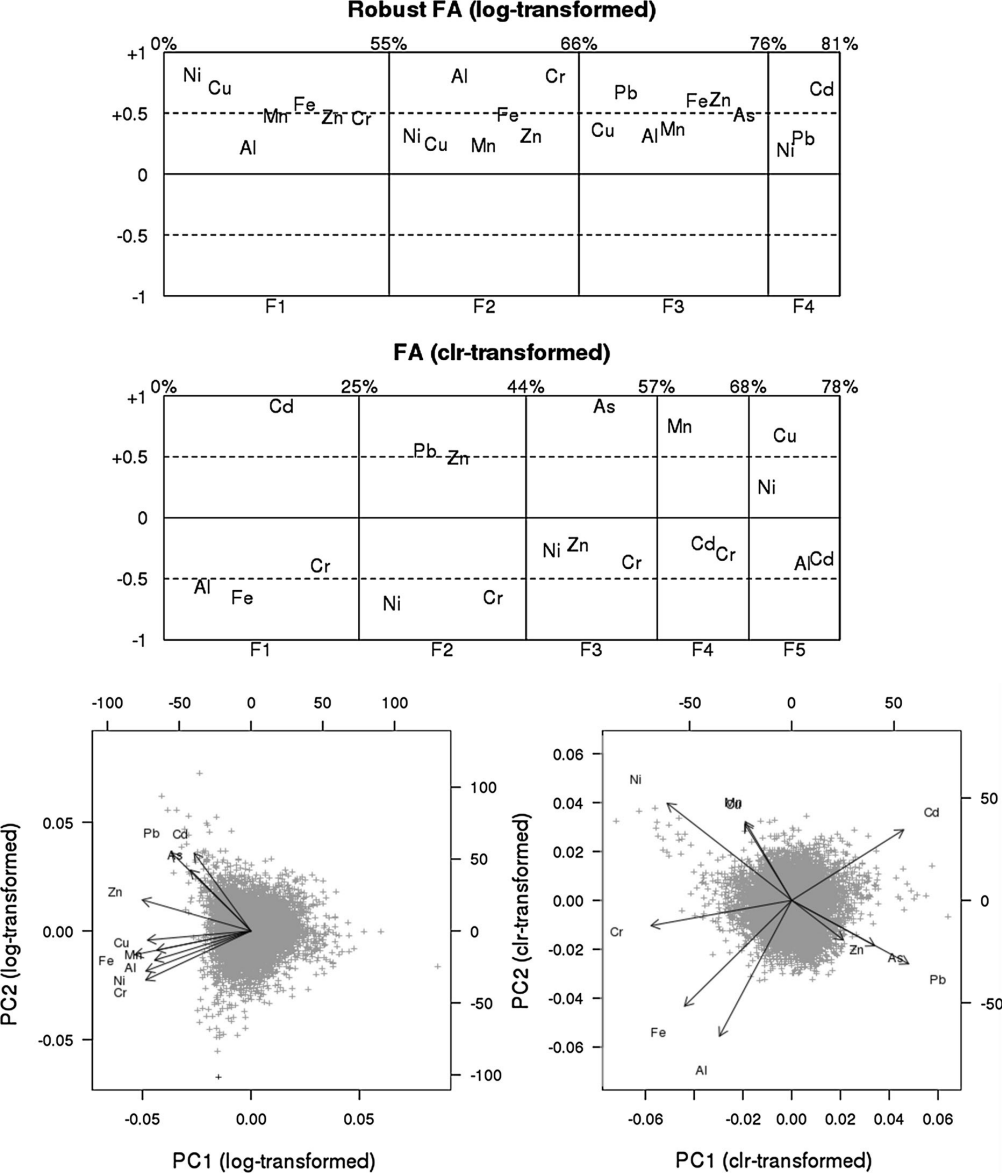
**a** Factor loading plots for the log-transformed and centred logratio-transformed (clr-transformed) four- and five-factor models (PFA and varimax rotation). **b** Biplot of PC1 versus PC2 (Principal Components analysis) of the log-transformed (*left*) and clr-transformed (*right*) loadings (*arrows*) and scores (*data points*)

Figure 1a shows the factor loading plots for the log-transformed and clr-transformed four- and five-factor models, respectively (PFA and varimax rotation). The position of the element names in the plot reflects the loading of that element on the different factors. In addition, the percentages at the top of the plots display the cumulative explained percentage of total variability. The scale on the horizontal axis is in accordance with the relative amount of variability explained by each single factor (Filzmoser et al. 2009). This figure gives an idea of the significance/composition of each factor. The comments make reference to items with factor scores ≥|0.4|. In the *log-transformed factorial analysis*, the respective factors were defined by the following positive loadings: F1 by Ni, Cu, Fe and Mn; F2 by Cr, Al and Fe; F3 by Pb, Zn, Fe and As; and F4 by Cd. In the *clr-transformed factorial analysis,* in contrast, the factors (sign changed) were defined as follows: F1 by a combination of positive loadings of Cd and negative loadings of Fe and Al; F2 by Pb, Zn and negative loadings of Ni and Cr; and F3, F4 and F5 by positive loadings of As, Mn and Cu, respectively. There was negligible correspondence between the factors of the two transformations. In a biplot, Fig. 1b shows the important change brought about by clr transformation.

Tables 1 and 2, with the log-transformed and clr-transformed data, respectively, show the RRs with 95% credibility intervals of the four and five score loads for the tumours analysed, by sex. Results are shown with adjustment for potential socio-demographic confounders. Results in that the 95% CI not including the unity, are in bold.

**Table 1:** Estimates of the effect [RR and 95% credibility interval (CI)] of factors corresponding to score loads from principal factor analysis, on mortality due to different tumour types, by sex for the log-transformed data analysis adjusted for socio-demographic variables

Cancer site		Men			Women		
		RR	95%	CI	RR	95%	CI
Lung	F1	NA	NA	NA	1.026	0.980	1.069
Buccal cavity and pharynx	F1	0.996	0.948	1.044	0.997	0.939	1.063
Oesophagus	F1	0.956	0.899	1.001	1.013	0.908	1.104
Stomach	F1	1.007	0.963	1.046	0.995	0.952	1.039
Pancreas	F1	1.002	0.971	1.042	0.981	0.947	1.020
Colorectal	F1	1.013	0.986	1.042	1.011	0.987	1.042
Breast	F1				1.004	0.976	1.030
Prostate	F1	1.000	0.975	1.030			
Bladder	F1	0.990	0.957	1.030	0.995	0.950	1.044
Kidney	F1	1.011	0.969	1.061	0.993	0.941	1.047
Brain	F1	1.008	0.970	1.043	1.026	0.986	1.078
NHL	F1	0.996	0.949	1.041	1.016	0.976	1.057
Leukaemias	F1	0.998	0.965	1.033	0.990	0.955	1.032
Lung	F2	**0.952**	**0.918**	**0.989**	1.016	0.946	1.075
Buccal cavity and pharynx	F2	**0.907**	**0.846**	**0.965**	1.089	0.994	1.188
Oesophagus	F2	0.961	0.899	1.029	1.148	0.838	1.357
Stomach	F2	0.990	0.941	1.042	0.996	0.937	1.056
Pancreas	F2	1.019	0.975	1.067	0.994	0.938	1.042
Colorectal	F2	0.990	0.955	1.027	0.975	0.942	1.009
Breast	F2				0.978	0.943	1.013
Prostate	F2	1.010	0.975	1.048			
Bladder	F2	0.975	0.928	1.021	0.998	0.935	1.072
Kidney	F2	0.972	0.916	1.036	1.008	0.932	1.085
Brain	F2	0.978	0.933	1.034	1.027	0.969	1.096
NHL	F2	0.961	0.900	1.021	1.045	0.982	1.104
Leukaemias	F2	0.993	0.930	1.040	1.013	0.964	1.075
Lung	F3	**1.123**	**1.084**	**1.146**	1.037	0.980	1.077
Buccal cavity and pharynx	F3	**1.062**	**1.015**	**1.109**	1.025	0.970	1.089
Oesophagus	F3	1.063	0.992	1.113	**1.121**	**1.026**	**1.225**
Stomach	F3	1.008	0.980	1.048	0.977	0.939	1.022
Pancreas	F3	1.019	0.980	1.049	1.017	0.983	1.050
Colorectal	F3	0.991	0.966	1.017	0.987	0.966	1.012
Breast	F3				1.004	0.977	1.027
Prostate	F3	0.998	0.973	1.024			
Bladder	F3	0.990	0.953	1.022	0.972	0.925	1.011
Kidney	F3	1.014	0.973	1.054	1.017	0.964	1.066
Brain	F3	0.999	0.968	1.034	0.988	0.945	1.025
NHL	F3	1.019	0.969	1.060	**1.044**	**1.002**	**1.081**
Leukaemias	F3	**1.048**	**1.015**	**1.080**	1.018	0.985	1.056
Lung	F4	0.993	0.950	1.032	1.009	0.953	1.068
Buccal cavity and pharynx	F4	1.052	0.989	1.121	1.053	0.977	1.139
Oesophagus	F4	1.023	0.962	1.095	1.122	0.975	1.445
Stomach	F4	0.977	0.929	1.030	1.018	0.962	1.082
Pancreas	F4	1.029	0.988	1.083	0.998	0.954	1.048
Colorectal	F4	1.036	0.999	1.072	0.990	0.958	1.023
Breast	F4				0.996	0.964	1.036
Prostate	F4	1.014	0.979	1.050			
Bladder	F4	1.044	0.994	1.095	1.051	0.995	1.120
Kidney	F4	**1.061**	**1.006**	**1.133**	1.005	0.944	1.082
Brain	F4	1.035	0.993	1.084	**1.110**	**1.054**	**1.171**
NHL	F4	1.016	0.963	1.085	0.981	0.934	1.036
Leukaemias	F4	0.997	0.957	1.048	0.981	0.936	1.027

Results that do not include the unity in the 95% credibility interval (statistically significants) are in bold.

*F1*: Ni, Cu, Fe, Zn, Cr; *F2*: Al, Cr, Fe; *F3*: Pb, Zn, Fe, As; *F4*: Cd

**Table 2:** Estimates of the effect [RR and 95% credibility interval (CI)] of factors corresponding to score loads from principal factor analysis, on mortality due to different tumour types, by sex for the clr-transformed data analysis adjusted for socio-demographic variables

Cancer site			Men			Women		
			RR	95%	CI	RR	95%	CI
Lung	F1	Cd	1.003	0.958	1.044	0.948	0.897	1.007
Buccal cavity and pharynx	F1		**1.099**	**1.033**	**1.181**	0.987	0.908	1.065
Oesophagus	F1		1.052	0.982	1.126	0.978	0.851	1.250
Stomach	F1		0.981	0.931	1.041	1.001	0.944	1.070
Pancreas	F1		1.014	0.973	1.064	1.010	0.965	1.064
Colorectal	F1		1.023	0.984	1.060	0.999	0.968	1.040
Breast	F1					1.026	0.992	1.064
Prostate	F1		1.002	0.966	1.041			
Bladder	F1		**1.057**	**1.008**	**1.110**	1.043	0.987	1.111
Kidney	F1		1.049	0.992	1.110	1.006	0.943	1.099
Brain	F1		1.018	0.976	1.066	**1.081**	**1.024**	**1.142**
NHL	F1		1.027	0.971	1.093	0.980	0.927	1.030
Leukaemias	F1		0.988	0.934	1.099	0.998	0.945	1.042
Lung	F2	Pb	**1.038**	**1.009**	**1.069**	1.017	0.967	1.056
Buccal cavity and pharynx	F2		**1.062**	**1.019**	**1.112**	1.008	0.954	1.062
Oesophagus	F2		**1.065**	**1.013**	**1.112**	**1.093**	**1.013**	**1.202**
Stomach	F2		1.019	0.984	1.072	0.990	0.950	1.030
Pancreas	F2		1.002	0.974	1.034	1.013	0.981	1.051
Colorectal	F2		0.994	0.967	1.019	0.987	0.966	1.011
Breast	F2					1.001	0.981	1.026
Prostate	F2		0.997	0.973	1.024			
Bladder	F2		0.994	0.962	1.029	0.982	0.93	1.021
Kidney	F2		1.007	0.969	1.048	1.016	0.972	1.065
Brain	F2		0.991	0.961	1.023	0.982	0.945	1.017
NHL	F2		1.02	0.975	1.061	1.007	0.973	1.046
Leukaemias	F2		**1.046**	**1.013**	**1.076**	1.022	0.986	1.053
Lung	F3	As	1.030	0.995	1.065	1.036	0.984	1.089
Buccal cavity and pharynx	F3		1.054	0.996	1.118	1.007	0.933	1.095
Oesophagus	F3		1.017	0.957	1.077	**0.891**	**0.798**	**1.000**
Stomach	F3		1.028	0.983	1.077	1.017	0.966	1.073
Pancreas	F3		1.012	0.970	1.052	1.004	0.959	1.046
Colorectal	F3		1.034	0.993	1.066	1.022	0.993	1.054
Breast	F3					0.999	0.962	1.028
Prostate	F3		1.002	0.967	1.034			
Bladder	F3		1.037	0.995	1.082	1.019	0.963	1.082
Kidney	F3		1.047	0.992	1.103	0.995	0.929	1.059
Brain	F3		**1.064**	**1.016**	**1.108**	1.027	0.968	1.079
NHL	F3		1.026	0.972	1.082	1.016	0.969	1.072
Leukaemias	F3		1.007	0.962	1.047	0.994	0.950	1.039
Lung	F4	Mn	0.996	0.968	1.040	0.979	0.934	1.030
Buccal cavity and pharynx	F4		0.967	0.921	1.028	**0.906**	**0.844**	**0.959**
Oesophagus	F4		0.976	0.930	1.036	0.926	0.840	1.044
Stomach	F4		**1.058**	**1.012**	**1.099**	1.008	0.959	1.055
Pancreas	F4		0.997	0.962	1.031	0.986	0.951	1.026
Colorectal	F4		1.01	0.981	1.038	1.018	0.983	1.044
Breast	F4					1.018	0.987	1.046
Prostate	F4		1.017	0.989	1.047			
Bladder	F4		0.998	0.962	1.039	0.963	0.913	1.013
Kidney	F4		1.004	0.951	1.050	1.013	0.953	1.075
Brain	F4		1.036	0.996	1.078	0.960	0.917	1.008
NHL	F4		0.976	0.931	1.028	1.017	0.968	1.062
Leukaemias	F4		1.003	0.965	1.041	1.007	0.967	1.052
Lung	F5	Cu	**1.032**	**1.000**	**1.061**	**1.053**	**1.010**	**1.101**
Buccal cavity and pharynx	F5		**1.093**	**1.043**	**1.150**	1.009	0.948	1.081
Oesophagus	F5		0.993	0.944	1.043	1.031	0.932	1.134
Stomach	F5		0.99	0.954	1.032	0.984	0.944	1.035
Pancreas	F5		0.99	0.959	1.026	0.988	0.953	1.038
Colorectal	F5		1.024	0.997	1.052	1.016	0.990	1.041
Breast	F5					1.000	0.975	1.028
Prostate	F5		0.981	0.953	1.008			
Bladder	F5		1.001	0.968	1.043	0.998	0.947	1.047
Kidney	F5		1.029	0.986	1.088	0.994	0.944	1.054
Brain	F5		0.993	0.956	1.031	1.010	0.966	1.056
NHL	F5		1.033	0.984	1.081	0.987	0.947	1.032
Leukaemias	F5		**1.039**	**1.005**	**1.078**	0.995	0.953	1.032

Results that do not include the unity in the 95% credibility interval (statistically significants) are in bold.

*F1*: Cd, -Fe, -Al; *F2*: -Ni, -Cr, Pb, Zn; *F3*: As; *F4*: Mn; *F5*: Cu

In the case of the *log-transformed* variable with the four factors adjusted for socio-demographic variables, Table 1 shows excess mortality among *men* for F3 (Pb, Zn, Fe, As) in cancers of the lung, buccal cavity and pharynx, and leukaemias. The contrary (a protective effect, in which the upper limit of the credibility interval is below unity) was found for F2 (Al, Cr, Fe) in cancers of the lung, buccal cavity and pharynx. Among women, excess mortality was detected for F3 (Pb, Zn, Fe, As) in oesophageal cancer and LNH, and for F4 (Cd) in brain cancer. No protective effect with respect to mortality was detected in women.

Where there are negative factor loadings, interpretation of results is complicated by the fact that the factors refer to relative combinations of elements. Since elements with negative factor loadings were predominant in this analysis, the sign was changed to make interpretation of the results easier. In the analysis of the clr-transformed variable adjusted for socio-demographic variables shown in Table 2, excess risk was detected for factors F2 (–Ni –Cr Pb Zn) and F4 (Mn) in tumours of the digestive system (oesophagus and stomach), F1 (Cd) in cancer of bladder, F3 (As) in brain cancer, and F2 (–Ni –Cr Pb Zn) and F5 (Mn) in leukaemias. *Women* registered a lower mortality due to cancers of the buccal cavity and pharynx, and oesophagus for factors F4 (Mn) and F3 (As), respectively. Excess mortality was detected in lung [F5 (Cu)], brain [F1 (Cd)] and oesophageal cancer [F2 (–Ni –Cr Pb Zn)]. Excess mortality in both sexes was only observed in oesophageal cancer for F2 and lung cancer for F5.

The comparison of the two analyses (log transformation vs. clr transformation) was complicated by the different meaning of the factors and the factor loadings of different sign in the clr-transformed analysis. Any similarities attributable to the presence of lead would be represented by factor F3 (0.678) in the log-transformed analysis and by factor F2 (0.554) in the clr-transformed analysis. Hence, whereas excess mortality was detected by the log-transformed analysis for cancers of the lung, buccal cavity and pharynx, and leukaemias in men, and for oesophageal cancer and NHL in women, this was detected by the clr-transformed analysis for the same tumours plus oesophageal cancer in men.

In the case of Cd, the possible correspondence would be between factor F4 in the log-transformed and F1 in the clr-transformed analyses, i.e. while the log-transformed analysis showed excess mortality due to kidney cancer in men and brain cancer in women, the clr-transformed analysis showed excess mortality due to cancers of the buccal cavity, pharynx and bladder in men and brain cancer in women.

In both analyses, stress should be laid on the importance of adjustment for socio-demographic variables, since a considerable reduction in the effect (confounding effect) is evident in many of the outcomes of the associations in the crude analyses (see Supplementary material).

## Discussion

The results of this ecological mortality analysis show that soil composition could have an influence on the spatial distribution and mortality patterns of cancer. The original soil composition data were transformed by logratio transformation and subjected to factorial analysis, with the resulting factor scores being included as explanatory variables of exposure in the spatial regression models. The analysis adjusted for socio-demographic variables shows a number of associations not accounted for by random chance. These associations are in both directions, i.e. positive (risk factor) and negative (protective factor), as are the factor loadings. Among men, excess mortality was observed for tumours of the digestive system in soils with higher Pb, Zn, Mn, Cu and Cd concentrations, bladder cancer in soils with higher Cd concentrations, and brain cancer in soils with As. Among women, excess mortality was observed for brain tumours in the case of factor F1 (Cd) and lung cancer in the case of F5 (Cu).

Male mortality was higher than expected both for brain cancer, due to the presence of the inverted factor F3 -characterised by a high As concentration (loading of 0.917), and for cancer of buccal cavity and pharynx, and leukaemias, due to the presence of F5 (Cu). Similarly, among women higher concentrations of Cd could be associated with brain cancer and those of Cu with lung cancer. The results for F2 would appear to be better explained by the presence of lead. In the case of this factor, excess risk was observed for cancers of the lung, buccal cavity, oesophagus and leukaemias, though these same excesses were not in evidence among women.

The findings for F3 (As) in respect of oesophageal cancer among women (lower mortality than expected) would seem to contradict those of previous studies (Núñez et al. 2016), though this might be determined by the low presence of Cr in this factor. However, a review of F3 (As) outcomes shows the RRs to be higher than 1 in women for cancers of the lung, buccal cavity and pharynx, stomach, pancreas, colorectal, bladder, brain and NHL (all except oesophagus, kidney, and leukaemias), and higher than 1 in men for all the tumours studied, though without statistical significance being reached in any case. These results are in line with those published by previous studies (Núñez et al. 2016).

There is sufficient evidence in humans and experimental animals of the carcinogenicity of cadmium and cadmium compounds. Indeed, exposure to cadmium and cadmium compounds causes cancer at several sites (Straif et al. 2009). F2 factor is represented by lead concentrations, and associations were found for cancers of lung, buccal cavity and pharynx, and leukaemias in men, and for oesophageal cancer in both sexes. Associations between lead and cancer have been reported by other types of studies in the case of cancer of the stomach (Zhao et al. 2014) and pancreas (Amaral et al. 2012). The IARC classifies inorganic lead compounds as probably carcinogenic to humans (Group 2A), and lead exposure is known to increase the risk of lung, stomach, and bladder cancer (IARC Working Group et al. 2006). With respect to F5, represented by Cu, an association was found with lung cancer in both sexes, and with cancer of buccal cavity and pharynx, and leukaemias in men. Nevertheless, neither the EPA nor the IARC (Group 3) classifies copper as a human carcinogen because there are no suitable human or animal cancer studies.

With respect to compositional analysis, for multivariate data the effects of “closure” can be overcome by applying a suitable logratio transformation (i.e. clr transformation) (Aitchison 2003; Reimann et al. 2011). When carrying out principal component or factor analysis, the effect of opening the data is conspicuous on a biplot (Fig. 1b). When carrying out principal components or factor analysis, the effect of opening the data is conspicuous on a biplot (Fig. 1b). Only logratio-transformed data provide information about the true relationships between the variables, relationships which are independent of the total concentrations of the elements (Reimann et al. 2012). The use of log transformation made it possible to compare the differences and concordances between the results of the two analyses. Examples in epidemiology of compositional data analysis are to be found in recent nutrition and human microbiome studies, though there is an acknowledged lack of sufficient methodological development in this sphere (Leite 2014; Tsilimigras and Fodor 2016).

Furthermore, it is important to highlight the fact that data transformation and factorial analysis pose difficulties when it comes to interpretation of results in terms of population risk. Rather than referring to specific elements or to relative risks for a category of exposure to an element, the results instead refer to their associations or topsoil composition patterns.

Outliers were not eliminated from this analysis. Even so, we verified that elimination of outliers in factorial analysis does not alter the composition of the factors obtained (results not shown) and, furthermore, that the analysis procedure implies a smoothing of the estimate of the level of each element in each town similar to that obtained by a kriging interpolation.

Analysis of point–point misaligned data (Finley et al. 2014; Lindgren and Rue 2015) has provided a viable solution for epidemiological analysis in the form of SPDE and R-INLA. INLA, combined with the SPDE approach (Lindgren et al. 2011), can easily accommodate all types of geographically referenced data, including areal, geostatistical and spatial point process data (Blangiardo and Cameletti 2015). The software enables one to construct the triangulation mesh and programme the joint modelling of the sampling points and mortality with BYM autoregressive models. The computation times, though very long, are, nevertheless, acceptable using high-performance equipment, with R-INLA itself ensuring the parallelisation of the process by using all available processors for the purpose.

The weaknesses of this study are those inherent in ecological mortality studies and the use of data aggregated by town. Little or nothing is known about many potential aspects of exposure stemming from soil composition, individual lifestyles and variables of great importance in cancer, such as smoking. Accordingly, the assumption inherent in this study, i.e. that the population consumes local products, is acceptable for small towns but not for large cities where the bulk of family food buying currently takes place at major shopping malls. In view of the long latency periods in cancer, however, it can be assumed that in past decades, the consumption of local products was more generalised.

To our knowledge, there are no other studies comparable to ours in terms of dimension and scope. This study covered mainland Spain and contains soil concentration measurements of 10 elements (heavy metals and metalloids) for a mesh of >13,000 sampling points. It also includes cancer mortality data in over 8000 towns across a 10-year study period, recording a total of 861,440 deaths due to the tumours analysed. For statistical treatment purposes, use was made of hierarchical models, with spatial components (Besag et al. 1991) being adjusted by R-INLA (Lindgren and Rue 2015). In these models, the risk of ecological fallacy was minimised by using a very fine spatial scale and making no inferences at an individual level (Clayton et al. 1993; Cressie et al. 2009). Moreover, to account for the spatial interpolation error in the inference, a multivariate model for spatially misaligned data was used (the set of observed locations for the explanatory variable is not identical to that for the response variable) (Cameletti et al. 2013). The inference in this model was performed by means of the SPDE approach (Lindgren et al. 2011), thus making it computationally feasible and efficient.

By way of conclusion, attention should be drawn to the fact that these new results support the relative role which topsoil composition may play in the frequency and geographical distribution of cancer, and to the importance of taking into account the compositional nature of the data in the analysis, despite the difficulties of interpretation of results that this generates.

## Electronic supplementary material

Below is the link to the electronic supplementary material.

Supplementary material 1 (PDF 145 kb)
