# Supplementary material for: Compositional analysis of topsoil metals and its associations with cancer mortality using spatial misaligned data
Source: Environ Geochem Health. 2017 Feb 2;40(1):283–94. doi: 10.1007/s10653-016-9904-3 (PMC5797570; doi:10.1007/s10653-016-9904-3)
Supplement: Supplementary file 1 — Supplementary material 1 (PDF 145 kb) [file 10653_2016_9904_MOESM1_ESM.pdf]

Table S1. Number of deaths by different studied tumours produced in continental Spain between the years 1999-2008.

Cancer site	ICD-9	ICD-10	Deaths Men	Deaths Women	Total
Lung	C33-C34	162	155142	21657	176799
Buccal cav. and pharynx	C00-C14	140-149	16274	3761	20035
Oesophagus	C15	150	14287	2228	16515
Stomach	C16	151	34679	21692	56371
Pancreas	C25	157	22458	19816	42274
Colorectal	C18-C21	153-154,159.0	68353	51310	119663
Breast	C50	174	-	54887	54887
Prostate	C61	185	52528	-	52528
Bladder	C67	188	33632	7212	40844
Kidney	C64-C66,C68	189	11509	5854	17363
Brain	C71	191	12371	9881	22252
NHL	C82-C85,C96	200,202	11706	10499	22205
Leukemias	C91-C95	204-208	15861	12376	28237

Table S2. Estimates of the effect (RR and 95% credibility interval (CI)) of factors corresponding to score loads from principal factor analysis, on mortality due to different tumour types, by sex. The table shows the results of the **log-transformed** data analysis unadjusted and adjusted for socio-demographic variables.

		Men						Women					
		Unadj			Adjus			Unadj			Adjus		
**Cancer site**		RR	95%	CI	RR	95%	CI	RR	95%	CI	RR	95%	CI
Lung	F1	NA	NA	NA	NA	NA	NA	1.103	1.077	1.157	1.026	0.98	1.069
Buccal cavity and pharynx	F1	0.934	0.892	0.958	0.996	0.948	1.044	0.991	0.934	1.054	0.997	0.939	1.063
Oesophagus	F1	0.935	0.891	0.969	0.956	0.899	1.001	1.023	0.933	1.137	1.013	0.908	1.104
Stomach	F1	0.984	0.934	1.054	1.007	0.963	1.046	0.957	0.904	1.011	0.995	0.952	1.039
Pancreas	F1	0.978	0.949	1.017	1.002	0.971	1.042	0.945	0.919	0.981	0.981	0.947	1.02
Colorectal	F1	1.069	1.053	1.101	1.013	0.986	1.042	0.973	0.954	1.002	1.011	0.987	1.042
Breast	F1							0.97	0.931	1.004	1.004	0.976	1.03
Prostate	F1	1.007	0.964	1.104	1	0.975	1.03						
Bladder	F1	0.93	0.896	0.947	0.99	0.957	1.03	1.062	0.934	1.352	0.995	0.95	1.044
Kidney	F1	1.007	0.962	1.057	1.011	0.969	1.061	0.98	0.929	1.038	0.993	0.941	1.047
Brain	F1	1	0.967	1.039	1.008	0.97	1.043	1.023	0.976	1.066	1.026	0.986	1.078
NHL	F1	0.919	0.866	0.967	0.996	0.949	1.041	1.055	1.012	1.088	1.016	0.976	1.057
Leukemias	F1	0.984	0.96	1.012	0.998	0.965	1.033	1	0.954	1.096	0.99	0.955	1.032
Lung	F2	0.85	0.823	0.871	0.952	0.918	0.989	1.151	1.113	1.235	1.016	0.946	1.075
Buccal cavity and pharynx	F2	0.797	0.752	0.844	0.907	0.846	0.965	1.102	1.01	1.197	1.089	0.994	1.188
Oesophagus	F2	1.077	1.004	1.136	0.961	0.899	1.029	1.249	1.087	1.395	1.148	0.838	1.357
Stomach	F2	0.939	0.876	0.988	0.99	0.941	1.042	0.928	0.875	0.987	0.996	0.937	1.056
Pancreas	F2	1.044	0.982	1.092	1.019	0.975	1.067	1.016	0.928	1.074	0.994	0.938	1.042
Colorectal	F2	0.915	0.876	0.935	0.99	0.955	1.027	0.941	0.914	0.979	0.975	0.942	1.009
Breast	F2							1.01	0.944	1.06	0.978	0.943	1.013
Prostate	F2	1.014	0.965	1.05	1.01	0.975	1.048						
Bladder	F2	0.904	0.859	0.928	0.975	0.928	1.021	1.038	0.961	1.1	0.998	0.935	1.072
Kidney	F2	0.97	0.905	1.03	0.972	0.916	1.036	0.998	0.92	1.073	1.008	0.932	1.085
Brain	F2	0.986	0.935	1.033	0.978	0.933	1.034	1.028	0.97	1.096	1.027	0.969	1.096
NHL	F2	0.915	0.868	0.977	0.961	0.9	1.021	1.054	0.935	1.126	1.045	0.982	1.104
Leukemias	F2	1.038	0.989	1.075	0.993	0.93	1.04	1.021	0.968	1.072	1.013	0.964	1.075
Lung	F3	1.082	1.071	1.106	1.123	1.084	1.146	1.119	1.092	1.165	1.037	0.98	1.077
Buccal cavity and pharynx	F3	1.137	1.097	1.22	1.062	1.015	1.109	1.053	0.985	1.112	1.025	0.97	1.089
Oesophagus	F3	1.099	1.069	1.149	1.063	0.992	1.113	1.175	1.089	1.292	1.121	1.026	1.225
Stomach	F3	1.097	0.999	1.297	1.008	0.98	1.048	0.957	0.917	0.987	0.977	0.939	1.022
Pancreas	F3	1.048	1.018	1.08	1.019	0.98	1.049	1.041	1.003	1.074	1.017	0.983	1.05
Colorectal	F3	1.055	1.038	1.08	0.991	0.966	1.017	1.03	1.004	1.049	0.987	0.966	1.012
Breast	F3							1.063	1.038	1.116	1.004	0.977	1.027
Prostate	F3	1.048	1.022	1.065	0.998	0.973	1.024						
Bladder	F3	1.048	1.032	1.082	0.99	0.953	1.022	1.019	0.971	1.058	0.972	0.925	1.011
Kidney	F3	1.036	0.993	1.079	1.014	0.973	1.054	1.025	0.967	1.077	1.017	0.964	1.066
Brain	F3	1.001	0.913	1.048	0.999	0.968	1.034	1.015	0.973	1.049	0.988	0.945	1.025
NHL	F3	1.087	1.058	1.133	1.019	0.969	1.06	1.082	1.046	1.131	1.044	1.002	1.081
Leukemias	F3	1.056	1.024	1.087	1.048	1.015	1.08	1.026	0.994	1.06	1.018	0.985	1.056
Lung	F4	1.111	1.089	1.144	0.993	0.95	1.032	1.155	1.114	1.24	1.009	0.953	1.068
Buccal cavity and pharynx	F4	1.125	1.088	1.195	1.052	0.989	1.121	1.062	0.981	1.143	1.053	0.977	1.139
Oesophagus	F4	1.163	1.112	1.222	1.023	0.962	1.095	1.1	0.958	1.278	1.122	0.975	1.445

Stomach	F4	0.938	0.878	0.992	0.977	0.929	1.03	1.095	1.031	1.202	1.018	0.962	1.082
Pancreas	F4	1.091	1.044	1.16	1.029	0.988	1.083	1.039	0.964	1.097	0.998	0.954	1.048
Colorectal	F4	1.103	1.081	1.145	1.036	0.999	1.072	1.025	0.983	1.067	0.99	0.958	1.023
Breast	F4							1.107	1.043	1.145	0.996	0.964	1.036
Prostate	F4	1.029	0.993	1.067	1.014	0.979	1.05						
Bladder	F4	1.131	1.128	1.59	1.044	0.994	1.095	1.084	1.02	1.161	1.051	0.995	1.12
Kidney	F4	1.117	1.055	1.196	1.061	1.006	1.133	1.042	0.975	1.152	1.005	0.944	1.082
Brain	F4	1.057	1.015	1.108	1.035	0.993	1.084	1.12	1.061	1.184	1.11	1.054	1.171
NHL	F4	1.114	1.065	1.191	1.016	0.963	1.085	1.07	1.015	1.116	0.981	0.934	1.036
Leukemias	F4	1.035	0.991	1.07	0.997	0.957	1.048	0.988	0.95	1.031	0.981	0.936	1.027

F1: Ni Cu Fe Zn Cr
F2: Al Cr Fe
F3: Pb Zn Fe As
F4: Cd

Table S3. Estimates of the effect (RR and 95% credibility interval (CI)) of factors corresponding to score loads from principal factor analysis, on mortality due to different tumour types, by sex. The table shows the results of the **clr-transformed** data analysis unadjusted and adjusted for socio-demographic variables.

			Men						Women					
			Unadj			Adjus			Unadj			Adjus		
**Cancer site**			RR	95%	CI	RR	95%	CI	RR	95%	CI	RR	95%	CI
Lung	F1	Cd	NA	NA	NA	1.003	0.958	1.044	0.919	0.867	0.944	0.948	0.897	1.007
Buccal cavity and pharynx	F1		1.147	1.107	1.219	1.099	1.033	1.181	0.952	0.88	1.036	0.987	0.908	1.065
Oesophagus	F1		1.111	1.069	1.186	1.052	0.982	1.126	0.889	0.79	0.98	0.978	0.851	1.25
Stomach	F1		0.952	0.877	1.042	0.981	0.931	1.041	1.06	1.013	1.128	1.001	0.944	1.07
Pancreas	F1		1.052	0.981	1.11	1.014	0.973	1.064	1.052	0.999	1.1	1.01	0.965	1.064
Colorectal	F1		1.099	1.074	1.148	1.023	0.984	1.06	1.042	0.995	1.081	0.999	0.968	1.04
Breast	F1								1.091	1.063	1.143	1.026	0.992	1.064
Prostate	F1		1.029	0.993	1.06	1.002	0.966	1.041						
Bladder	F1		1.167	1.131	1.217	1.057	1.008	1.11	1.054	0.955	1.127	1.043	0.987	1.111
Kidney	F1		1.065	1.002	1.164	1.049	0.992	1.11	1.034	0.971	1.111	1.006	0.943	1.099
Brain	F1		1.024	0.986	1.077	1.018	0.976	1.066	1.077	0.985	1.142	1.081	1.024	1.142
NHL	F1		1.138	1.082	1.25	1.027	0.971	1.093	0.93	0.892	0.974	0.98	0.927	1.03
Leukemias	F1		0.989	0.935	1.108	0.988	0.934	1.099	0.992	0.955	1.041	0.998	0.945	1.042
Lung	F2	Pb	1.069	1.053	1.101	1.038	1.009	1.069	1.117	1.081	1.199	1.017	0.967	1.056
Buccal cavity and pharynx	F2		1.103	1.077	1.153	1.062	1.019	1.112	1.051	0.996	1.096	1.008	0.954	1.062
Oesophagus	F2		1.094	1.062	1.144	1.065	1.013	1.112	1.083	1.004	1.208	1.093	1.013	1.202
Stomach	F2		1.052	1.007	1.114	1.019	0.984	1.072	0.96	0.923	1.008	0.99	0.95	1.03
Pancreas	F2		1.042	1.007	1.069	1.002	0.974	1.034	1.033	0.998	1.072	1.013	0.981	1.051
Colorectal	F2		1.095	1.054	1.198	0.994	0.967	1.019	1.032	1.004	1.052	0.987	0.966	1.011
Breast	F2								1.056	1.038	1.085	1.001	0.981	1.026
Prostate	F2		0.999	0.921	1.04	0.997	0.973	1.024						
Bladder	F2		1.076	1.057	1.115	0.994	0.962	1.029	1.024	0.977	1.062	0.982	0.93	1.021
Kidney	F2		1.038	0.996	1.083	1.007	0.969	1.048	1.031	0.986	1.092	1.016	0.972	1.065
Brain	F2		1.01	0.97	1.039	0.991	0.961	1.023	1.015	0.974	1.046	0.982	0.945	1.017
NHL	F2		1.083	1.047	1.13	1.02	0.975	1.061	1.052	1.013	1.083	1.007	0.973	1.046
Leukemias	F2		1.055	1.02	1.084	1.046	1.013	1.076	0.988	0.858	1.055	1.022	0.986	1.053
Lung	F3	As	1.082	1.057	1.147	1.03	0.995	1.065	1.239	1.177	1.364	1.036	0.984	1.089
Buccal cavity and pharynx	F3		1.165	1.097	1.228	1.054	0.996	1.118	0.924	0.876	0.996	1.007	0.933	1.095
Oesophagus	F3		0.936	0.893	0.993	1.017	0.957	1.077	0.913	0.818	1.016	0.891	0.798	1
Stomach	F3		1.042	0.952	1.101	1.028	0.983	1.077	1.057	0.974	1.12	1.017	0.966	1.073
Pancreas	F3		1.039	0.992	1.074	1.012	0.97	1.052	1.035	0.985	1.073	1.004	0.959	1.046
Colorectal	F3		1.097	1.076	1.137	1.034	0.993	1.066	1.05	1.014	1.076	1.022	0.993	1.054
Breast	F3								0.945	0.918	0.982	0.999	0.962	1.028
Prostate	F3		1.019	0.987	1.043	1.002	0.967	1.034						
Bladder	F3		1.059	1.036	1.107	1.037	0.995	1.082	1.025	0.964	1.09	1.019	0.963	1.082
Kidney	F3		1.047	0.99	1.111	1.047	0.992	1.103	0.981	0.916	1.047	0.995	0.929	1.059
Brain	F3		1.039	0.997	1.096	1.064	1.016	1.108	1.012	0.959	1.067	1.027	0.968	1.079
NHL	F3		1.07	0.992	1.134	1.026	0.972	1.082	1.06	1.006	1.103	1.016	0.969	1.072
Leukemias	F3		1.034	0.993	1.065	1.007	0.962	1.047	1.012	0.964	1.048	0.994	0.95	1.039
Lung	F4	Mn	0.914	0.885	0.93	0.996	0.968	1.04	0.904	0.86	0.926	0.979	0.934	1.03
Buccal cavity and pharynx	F4		0.857	0.825	0.89	0.967	0.921	1.028	0.892	0.828	0.942	0.906	0.844	0.959
Oesophagus	F4		0.908	0.861	0.941	0.976	0.93	1.036	0.914	0.823	1.014	0.926	0.84	1.044

Stomach	F4		1.051	1.02	1.094	1.058	1.012	1.099	1.039	0.97	1.091	1.008	0.959	1.055
Pancreas	F4		0.961	0.926	1	0.997	0.962	1.031	0.961	0.923	1.003	0.986	0.951	1.026
Colorectal	F4		0.938	0.91	0.952	1.01	0.981	1.038	0.961	0.939	0.994	1.018	0.983	1.044
Breast	F4								0.927	0.854	0.963	1.018	0.987	1.046
Prostate	F4		0.998	0.963	1.066	1.017	0.989	1.047						
Bladder	F4		0.882	0.842	0.907	0.998	0.962	1.039	0.951	0.893	1.002	0.963	0.913	1.013
Kidney	F4		0.975	0.926	1.021	1.004	0.951	1.05	0.993	0.935	1.053	1.013	0.953	1.075
Brain	F4		1.011	0.972	1.052	1.036	0.996	1.078	0.947	0.903	0.995	0.96	0.917	1.008
NHL	F4		0.881	0.812	0.924	0.976	0.931	1.028	0.952	0.92	0.998	1.017	0.968	1.062
Leukemias	F4		0.984	0.951	1.026	1.003	0.965	1.041	0.993	0.956	1.05	1.007	0.967	1.052
Lung	F5	Cu	1.12	1.101	1.15	1.032	1	1.061	1.118	1.091	1.171	1.053	1.01	1.101
Buccal cavity and pharynx	F5		1.152	1.119	1.21	1.093	1.043	1.15	1.05	0.986	1.103	1.009	0.948	1.081
Oesophagus	F5		1.065	0.996	1.164	0.993	0.944	1.043	0.841	0.57	1.14	1.031	0.932	1.134
Stomach	F5		0.981	0.937	1.054	0.99	0.954	1.032	0.972	0.922	1.05	0.984	0.944	1.035
Pancreas	F5		1.036	0.994	1.068	0.99	0.959	1.026	0.97	0.942	1.01	0.988	0.953	1.038
Colorectal	F5		1.073	1.058	1.104	1.024	0.997	1.052	1.073	1.038	1.096	1.016	0.99	1.041
Breast	F5								1.095	1.034	1.237	1	0.975	1.028
Prostate	F5		0.998	0.961	1.074	0.981	0.953	1.008						
Bladder	F5		1.073	1.052	1.116	1.001	0.968	1.043	0.975	0.81	1.066	0.998	0.947	1.047
Kidney	F5		1.066	1.006	1.19	1.029	0.986	1.088	1.005	0.952	1.069	0.994	0.944	1.054
Brain	F5		1.003	0.971	1.045	0.993	0.956	1.031	1.022	0.966	1.066	1.01	0.966	1.056
NHL	F5		1.158	1.056	1.399	1.033	0.984	1.081	1.043	0.997	1.077	0.987	0.947	1.032
Leukemias	F5		1.044	1.011	1.093	1.039	1.005	1.078	1.004	0.949	1.04	0.995	0.953	1.032

F1: Cd -Fe -Al
F2: -Ni -Cr Pb Zn
F3: As
F4: Mn
F5: Cu
